# Material Proximities and Hotspots: Toward an Anthropology of Viral Hemorrhagic Fevers

**DOI:** 10.1111/maq.12092

**Published:** 2014-04-21

**Authors:** Hannah Brown, Ann H Kelly

**Affiliations:** Department of Anthropology, Durham University; Department of Sociology, Philosophy and Anthropology, University of Exeter

**Keywords:** hotspot, viral hemorrhagic fevers, material proximities, animal studies, space

## Abstract

This article outlines a research program for an anthropology of viral hemorrhagic fevers (collectively known as VHFs). It begins by reviewing the social science literature on Ebola, Marburg, and Lassa fevers and charting areas for future ethnographic attention. We theoretically elaborate the hotspot as a way of integrating analysis of the two routes of VHF infection: from animal reservoirs to humans and between humans. Drawing together recent anthropological investigations of human–animal entanglements with an ethnographic interest in the social production of space, we seek to enrich conceptualizations of viral movement by elaborating the circumstances through which viruses, humans, objects, and animals come into contact. We suggest that attention to the material proximities—between animals, humans, and objects—that constitute the hotspot opens a frontier site for critical and methodological development in medical anthropology and for future collaborations in VHF management and control.

## Introduction

The problems that define the global health agenda are linked to transnational determinates: urbanization, migration, war, market integration, environmental degradation, and climate change. The scale and pace of these processes, their extraterritorial distribution, and political dislocation create the conditions for new and/or drug-resistant pathogenic strains, threatening explosive pandemics and persistent plagues (Farmer 1999; Herring and Swedlund 2010). Perhaps the most feared of these emerging diseases are viral hemorrhagic fevers (VHFs)—a broad class of zoonotic infections present in diverse settings. We focus on three VHFs found in sub-Saharan Africa: Ebola, Marburg, and Lassa fevers.^1^ These VHFs are highly pathogenic, contagious, and, according to post–September 11 U.S. policy, potential bioterrorist agents (Polesky and Bhatia 2003).^2^ Although few experts believe it likely that these viruses will trigger wide-scale outbreaks, the potential of VHFs to cross species boundaries with little warning continues to fuel apocalyptic visions of viruses raging from remote African villages to global metropolises, killing millions in a matter of days (Wolfe 2011).^3^

Despite the prominent place of VHF outbreaks in Africa in the popular imagination, Ebola, Marburg, and Lassa fevers have, in large part, eluded anthropological analysis. Arguably, the dearth of ethnographic data on VHFs is due to the challenges of conducting fieldwork in an epidemic—both in terms of the risk to the anthropologist and the coincidence entailed in “being there” when an outbreak occurs. As in other emergency settings, the urgency for immediate action to control VHFs runs counter to the demands of a time-consuming and slow-paced research methodology.

Similar investigative constraints inform the substance and scope of this article. Our arguments do not draw from ethnographic fieldwork during an outbreak but rather from the experience of devising a program for collaborative research into the dynamics and management of VHFs in sub-Saharan Africa. Our efforts to delineate an ethnographic contribution that would both support and extend the activities of our colleagues—biologists, ecologists, virologists, mathematical modelers, and public health experts—prompted reflection on the potential of our discipline to analytically cut across the diverse lives, materials, places, and practices that influence transmission dynamics. 4 This article develops those insights into a theoretical framework for future anthropological studies of VHFs.

Our argument proceeds by bringing together the extensive, but highly fragmented, social science literature on VHFs and examining these contributions alongside the few but influential anthropological investigations on VHF outbreaks. To date, what epidemiologists call secondary transmission (human to human) has received far greater empirical attention than primary transmission (animal to human); however, scholarship on either topic is ethnographically thin by anthropological standards. Synthesizing insights from a range of qualitatively inclined sources—social science journals, papers in agricultural studies, NGO reports, ecological surveys, public health guidelines, memoirs, and even brief asides in epidemiological articles—and critically reading them against relevant anthropological research offers a way of “thickening” existing literature.^5^ While not a substitute for ethnography, this review draws attention to the richness of existing knowledge when read across discipline and genre and highlights the possibilities it offers for future anthropological investigation.

Staking out that terrain is only a first step. To come to grips with the significance of viral encounters—their virulence and their social meaning—requires not simply more ethnography or a contextualization of existing knowledge but a reconceptualization of the anthropological field of analysis. Borrowing a term from disease ecology and building on anthropological efforts to describe the interactions between different diseases within specific environmental and socioeconomic contexts (e.g., Singer 2010), we offer the *hotspot* as a possible heuristic for analyzing the complex relationalities that drive VHF transmission. While VHF refers to a disease category, the hotspot speaks to the temporary convergence of rainfall, political designs, cat populations, armed conflict, economic strategies, agricultural techniques, built environments, and practices of care that create the conditions for disease communicability. The hotspot draws on an ecological imaginary that takes us past the singular moment of viral contact into ‘the assemblages of diseases’ and their attendant medical geographies (Audy 1954:962) shaped by diverse social and ecological contexts (e.g., Dunn and Janes 1986; Herring and Swedlund 2010) but remains anchored by an ethnographic preoccupation with the intimate textures of transmission. In short, as opposed to what Patricia Wald terms an “outbreak narrative” or “the formulaic plot that begins with the identification of emerging infection, [and] includes a discussion of the global networks throughout which it travels” (2008:2), the hotspot speaks to the mundane interactions that create the conditions of pathogenic possibility.

We elaborate this approach in five sections. We begin by summarizing existing anthropological research into VHFs and suggest areas for methodological development. We then briefly outline the virology of VHFs: the human–animal interactions that lead to what is referred to as “primary transmission” and the forms of contact between humans and between humans and fomites (inanimate objects or substances capable of transmitting the virus) that account for “secondary transmission.” The distinction between primary and secondary transmission cues the discussions in the third and fourth sections, where we situate these two routes in social space and in so doing hint at the possibility of a symmetrical analysis.

The fifth section explores the contours of an ethnography of the hotspot. We develop this term anthropologically by suggesting an attention to “material proximities” (Fontein 2011)—a focus that captures both the spatio–temporal heterogeneity and the socio–political substance of pathogenic spread. Our approach owes a significant debt to the work of anthropologists who have succeeded against the odds in conducting ethnographic research in an outbreak setting. The research framework we propose builds on their work to contribute to interdisciplinary efforts to produce “robust models that encompass the complex interface between pathogen biology, vectors and reservoir behavior” (Janes et al. 2012:1884–1885).^6^

## Repositioning Anthropology

Three anthropologists are notable for their work with communities with direct exposure to Ebola and Marburg: Barry and Bonny Hewlett and the French physician–anthropologist Alain Epelboin. The Hewletts’ work (e.g., 2008) helped reconfigure the WHO's approach to VHF management, underlining how ethnographic engagements can enhance containment efforts. For example, in Gulu, northern Uganda, they documented how indigenous Acholi responses to epidemics (*gemo*) involved forms of care-giving that curtailed the spread of Ebola and supported public health efforts (Hewlett and Amola 2003). Similarly, Epelboin (e.g., Epelboin and Formenty 2005) has worked with outbreak teams in a number of sites to make internationally led interventions more sensitive to local conditions. This involved taking into account indigenous burial practices, modes of greeting, and the symbolism attached to protective clothing and gear used during outbreaks.

These studies have not only succeeded in improving the effectiveness of VHF management but also in normalizing the inclusion of anthropological perspectives in public health interventions during an epidemic (Leach 2008:13–15). However, although ground-breaking, they confine the anthropological contribution to an unnecessarily narrow remit. The work focuses almost exclusively on interactions between humans and on the impact of cultural practices, such as caregiving, burial, and local etiologies on disease management. As a result, this body of research largely omits social engagements with material, institutional, and animal worlds. Such accounts reflect a lingering tradition in anthropology, particularly within the study of medicine and disease, to label only certain parts of human life as “social,” thus excluding critical dimensions of practice from ethnographic analysis (see Mol 2002). While understanding the social mores involved in caring for the sick or burying the dead can help explain—and even change—hazardous behaviors during an outbreak, circumscribing ethnography to what locals do or know limits the potential of ethnographic insights on broader relational contexts of transmission.^7^

Recent inquiries into the discourses, technologies, and institutional practices of biosecurity provide another entry point into the study of VHF. Informed by the work of Michel Foucault, this strand of research begins precisely with the technical and logistical dimensions of VHF response that a focus on traditional culture tends to bracket out (e.g., Bass 1998; Braun 2007; Briggs 2011; Leach 2008). These approaches provide considerable insight into the geopolitical underpinnings of VHF surveillance—how, for instance, the technologies and agendas of global health are shaped by the war on terror (Collier et al. 2004) or by changing imaginaries of “global health” (King 2002; Wald 2008). However, in training their analysis on the rhetorics and ideologies of “emergence” they do not offer much purchase on the everyday texture of VHF transmission.

In contrast, this article elaborates a concern with global political economy through an ethnographic engagement with intimate encounters. We employ the hotspot as an analytical tool to mark out sites where different variants of human–animal–nonhuman entanglements facilitate the movement of pathogens. In disease ecology, the notion of the hotspot has been developed as a way of interrogating the spatial heterogeneity of pathogen transmission, drawing attention to sites where transmission is amplified through particular characteristics of individuals, populations and/or environments within dense relational webs (DeGroote et al. 2008; Gatrell 2002; Meade and Emch 2010; Paull et al. 2012). An analysis of the hotspot foregrounds the varied and disproportionate nature of transmission by analyzing the role of “super spreaders”—the Typhoid Marys and Broad Street Pumps—within ecological contexts that transform disease dynamics and create the conditions for outbreak (Lloyd-Smith et al. 2005).

Our approach to VHF develops this concern with the multiple and intersecting spatio–temporal processes that shape transmission. But rather than model those dynamics or map assemblages, we seek to open them to ethnographic scrutiny. The analytical task is partly one of empirical extension: An anthropology of the hotspot investigates social practice within a series of biotic and material encounters—giving equal ethnographic weight, say, to experiences with illness and the construction of grain stores (a welcoming home for rats). The greater conceptual challenge is to do justice to the characteristics of the hotspot that defy scalar logics—the interpenetration of environmental transformation and immunological responses and the intricate and contingent assemblage of animal behaviors, food shortages, and global health policy (Hinchliffe 2009).

Studying the hotspot ethnographically locates transmission within dynamics that may include ecological abundance and economic scarcity, social proximity and administrative distance, convergence and fragmentation, excess and lack. But futher, as we describe in greater depth below, it shifts focus away from establishing causal patterns and instead to the persistent and shifting spatial, material, and historical co-presences that shape viral amplification. The hotspot does not provide the context of transmission, but is rather a “context in action” (Lezaun and Woolgar 2013).

Our theorization of the hotspot brings together the analytical advances of multi-species anthropology (e.g., Haraway 2008; Kirksey and Helmreich 2010; Raffles 2010), post-structuralist approaches to space (e.g., Low 1996; Massey 2005), and studies in material culture (e.g., Appadurai 1986; Miller 2005). Bringing together this suite of critical resources also provides an opportunity to revisit literature in the anthropology of environment, medical geography, and cultural ecology concerned with the spatial dimensions of human health and behavior (Dunn 1968; Meade 1977; Pavlovskii 1966). Although their foci differ, these scholars share a concern with the matrix of human and non-human relations that constitute social life and use these entanglements to trouble traditional categories of analysis. We draw on these varied intellectual projects to better apprehend the dynamics of VHF transmission.

## Problematic Proximities: Modes of VHF Transmission

There is reasonable consensus that fruit bats are reservoirs for Ebola and Marburg, while the multimammate rat carries Lassa. Outbreaks of Ebola and Marburg have generally involved hunting and butchering non-human primates, which, like humans, can become hosts for the virus. However, there have been cases of infection through handling other dead animals including antelopes and porcupines.^8^ In the case of Lassa, transmission tends to occur through touching surfaces contaminated with rodent saliva or excreta (Mills 2005; Stephenson et al. 1984; Ter Meulen et al. 1996). All three diseases can pass between humans through contact with bodily fluids or contaminated objects.^9^ However, many details of transmission cycles, including the varying contributions of hosts and vectors to the rate of pathogenic spread, remain unknown (Hugot et al. 2001; Wolfe et al. 2005).

VHF differ greatly in virulence. Roughly 80% of Lassa cases are asymptomatic; when symptoms do present, they tend to be severe, resulting in the death of roughly 15–20% of hospitalized cases. In the case of Marburg and the Ebola-Zaire strain, case-fatality rates can reach up to 90%. But because Lassa is endemic in populations, affecting as many as 500,000 annually, the annual mortality rate for Lassa is higher (Birmingham and Kenyon 2001).

**Figure 1:**
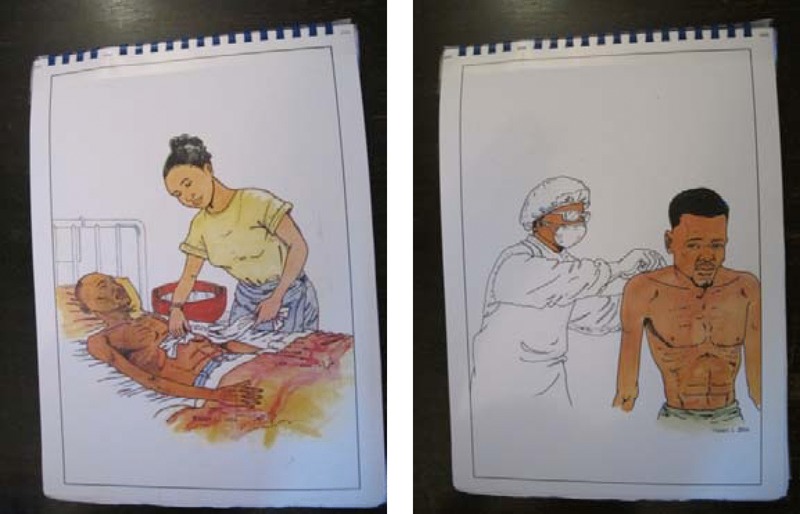
Images from a visual aid used in a public health campaign in DRC compare dangerous with protected caregiving of a patient with Ebola (LNSP/MSASF and France Cooperation N. d.).

In all three diseases, symptoms are diverse, ranging from high temperature, diarrhea, lethargy, shivering and aches, respiratory, and gastric problems. This variability poses diagnostic challenges; the frequency with which Ebola, Marburg, and Lassa are confused with malaria, influenza, or other diseases endemic to the area complicates prognosis (Kuhn 2008). As infection progresses, symptoms become more dramatic and include throat lesions, black vomit, deafness, and spontaneous internal and external bleeding. At this point, treatment for Ebola and Marburg is merely supportive; oral medication, rehydration, and nutritional supplements can alleviate some VHF-related symptoms, while psychosocial support can ease patient distress.^11^ Public health prevention programs revolve around reducing forms of human–animal contact viewed as potentially problematic (e.g., through rat control in sites where Lassa is endemic, wild animal surveillance networks, or the regulation of hunting) and developing strategies to curtail outbreaks through quarantine (see Figure 1).

## Viral Chatter: Animal Reservoirs and Viral “Spill Over”

VHF prevention hinges on reducing contact between humans and wildlife. A key epidemiological research strategy involves tracing the chain of clinical cases to an “index transmission event,” the moment when the virus spills over from its natural reservoir to the human host. Robust qualitative—to say nothing of ethnographic—evidence is rarely marshalled into those forensic accounts. However, human–animal relationships have long occupied a central place in anthropological inquiry (e.g., Douglas 1970; Evans-Pritchard 1940; Geertz 1994; Lévi-Strauss 1963; Rappaport 1968) and, with the emergence of multi-species ethnography as a mode of nature–culture critique, the discipline can clearly contribute much more towards illuminating the processes and pressures of coexistence (e.g., Haraway 2008; Kelly and Lezaun 2014; Kirksey and Helmreich 2010; Raffles 2010).

For the most part, studies that attend to the social dimensions of primary transmission focus on hunting. The expansion of the bushmeat trade has prompted extensive research within disease ecology and conservation biology exploring the role of large-scale political and environmental in shaping patterns of animal predation and consumption (Auzel and Wilkie 2000; Bifarin et al. 2008; Karesh and Noble 2009; Mickleburgh et al. 2009; Walsh et al. 2003).^11^ Extreme poverty and population displacement are generally understood as key drivers: With limited nutritional options, people eat whatever they can (Richmond and Baglole 2003).

The intertwined processes of resource extraction, political instability, and food scarcity clearly have a significant impact on why and how people hunt and eat potentially infectious animals (Leach and Hewlett 2010). A few qualitatively inclined studies have extended those insights to explore the social organization of bushmeat markets (Bowen-Jones et al. 2003; Tee et al. 2012) and the specific way ways in which animals are handled, priced, and sold (East et al. 2005; Mendelson et al. 2003; van Vliet and Mbazza 2011; Wilkie and Carpenter 1999). However, these studies are analytically constrained by their normative orientation: Because hunting is understood primarily as a risk to public health (and biodiversity), the symbolic significances, social relations, and affective dimensions of killing, butchering, and eating wild animals are generally left unexplored.

This is an area, however, where the anthropological record is considerably rich. Hunting and hunters are classic ethnographic subjects (Lévi-Strauss 1963; Myers 1988; Radcliffe-Brown 1965 [1952]) and, in recent years, sites of theoretical innovation (Descola 1996; Haraway 1989; Ingold 1988; Mullin 1999). While hunting was once held as an enduring artifact of emergent human civilization—a lens through which to explain gender relations, psychological proclivities, social organization, and the like—for contemporary anthropologists, hunting represents a profound (sometimes even poetic) engagement with the environment.

The study of hunting practices thus demands an integration of ecological, economic, symbolic, and post-structuralist perspectives (Giles-Vernick and Rupp 2006). Refusing to reduce animals to either sustenance or symbols (Shanklin 1985), the effort of these new anthropological theorizations is, rather, to explore the “consubstantiality” between animal behaviors and human culture (Croll et al. 1992). Scrambling conventional oppositions between traditional and modern, protection and predation, nature and culture, in these accounts, animals are understood as “selves” (Kohn 2007), “companions” (Haraway 2008), or “kin” (Fausto 2007) and hunting is construed as a form of sexual seduction (Willerslev 2004), “progeneration” (Brightman 1993) or a mode of ontological transformation (Brightman et al. 2014; Viveiros de Castro 1998).^12^

We cannot do justice to the theoretical diversity or conceptual thickness of this literature here, but let us point to two key contributions it can make to an anthropology of VHF. The first point relates to the inextricable and mutual entanglement of species: “In episodes of hunting” writes Tim Ingold, “the trails of human and animal cross and … each leaves bearing something of the substance of the other” (Ingold 2000:145). For ecologists, the interpenetration of human and animal bodies is obvious. However, when the social dimensions of transmission become the focus of study, the nonhuman forms of agency involved in pathogen exchange disappear. Understanding animals as co-participants rather than as vessels of disease could go a long way toward re-perceiving the varied encounters that lead to transmission.

Agustín Fuentes's 2006 pioneering work in ethnoprimatology is exemplary in this regard. In a comparative study of “monkey parks” in Bali and Gibraltar, Fuentes characterizes the potential for pathogenic exchange between humans and Macaques by filming their encounters. He analyzes their interactions in detail, noting detailed characteristics of both participants (age, gender, size for monkeys; for humans, clothing style and country of origin), what triggered these encounters (whether food was offered, and, if so, what kind), their emotional content (screaming, smiling, laughing), and their physical progression (did humans open their palms up or walk away; did macaques flash their eyelids or lunge and bite) (Fuentes 2006:885).

Fuentes (2006) further elaborates these descriptions of human/macaque behavior by recording the time of day and precise location in which they occurred as well as the humans’ and macaques’ relative positions in space. His research shows that while the broader contexts of interaction in these monkey parks may appear similar—the presence of European tourists, a domesticated macaque population—on closer inspection, striking differences emerge: In Bali, locals are more likely to be involved in the interactions; in Gibraltar, the most substantial interface is mediated by taxi and coach drivers who attempt to elicit responses from macaques for the sake of the tourists who have hired them.

Fuentes's work underscores the critical importance of attending in detail to the dynamics of interactional space. Transmission is the outcome of a process of engagement—monkeys do not just bite, they respond to cues from their human interlocutors (see Kohn 2007). Further, that his research takes place in a monkey park and not a forest frontier reminds us that our encounters with wildlife are not circumscribed to what is commonly understood as “the wild.” The current research bias toward studies of bushmeat reflects a limited imagination of interspecies traffic, one that fails to situate hunting within the multiple landscapes of everyday life. Although a few studies of Lassa fever have begun to pursue the links between the spatial distribution of infection and the quality of housing (e.g., Bonner et al. 2007; Kelly et al. 2013; Taylor et al. 2008), the modes in which animal reservoirs “become accustomed to the house”—even when the domestic may include the forest—have yet to be taken up as a site of social–scientific enquiry (Cassidy and Mullin 2007:5).

In addition to shifting the locus of research, work like Fuentes's transforms its topography. Mapping the dynamic zones of macaques–human overlap, Fuentes (2006) jettisons categories of natural, frontier, and domestic space. This relational approach to viral space is critical. First, because the conditions that trigger pathogenic exchange are multifaceted and persistent. Moreover, recent work in genetics suggest that zoonoses may only emerge and become established in human populations after repeated, unsuccessful, transmissions—or processes of what Wolfe et al. (2005) term “viral chatter.”^13^ Disease risk is not, then, “located,” in the sense of being a feature of a particular kind of place—forest frontiers, animal parks, and so on. Rather, it is locational and “ecosyndemic” (Singer 2010), arising from particular configurations of social, biotic, and material conditions, through “naturecultural corridors”—as Fuentes puts it (2010:609)—where potential pathogenic interactions are most dense.^14^

Consider the recent research by Leroy et al. (2009) into an Ebola outbreak in the DRC in the spring of 2007. In an effort to reconstruct the primary transmission event, the team began their search with the first confirmed Ebola death: a 55-year-old woman, who lived in one of an agglomeration of 10 villages that constituted the epicenter of the epidemic. They discovered that the village where she lived—like others in the region—had a “twin” village, about a three-to four-hour walk into the forest. Products of post-independence welfare policies, village settlements in the DRC were erected along the main road to facilitate access to public services. However, the population never fully relocated from their original dwellings, continuing to use their former homes as a base for hunting and agriculture. These twin villages also serve as the base for another population: each spring, a large number of bats (including two of the three species capable of carrying Ebola) come to feed on the palm tree nuts of an abandoned colonial-era palm oil plantation nearby.

**Figure 2:**
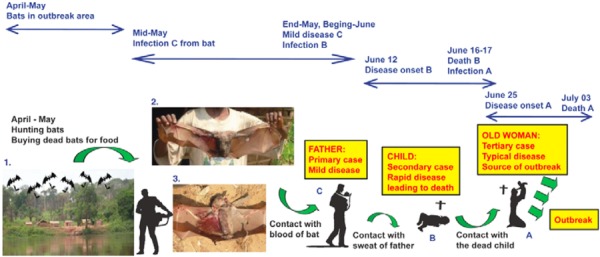
A flow chart tracing the chains of contact leading up to the “index transmission event’” (Leroy et al. 2009:727).

Capitalizing on this seasonal migration, hunters killed several bats on a daily basis, often with a shot gun (which can result in heavy bleeding) and sold the meat at a weekly market located in the villages near the road. Combing this information with the timing of the first death, Leroy et al. (2009) were able to hypothesize a chain of pathogenic connections from the bats to a young girl, whose father had purchased bat meat and had died from an unknown cause in early June, and ultimately, to the 55-year-old woman, who had helped the girl's grandparents wash her body in preparation for the funeral (see Figure 2).

This transmission narrative embodies what Fuentes (2009) describes as “niche construction”—the dynamic elaboration of mutual ecologies. Viral spread is not reducible to viral contact: Ebola emerges within a “meshwork” (Ingold 2008) of palm nuts, shotguns, national policies, and colonial pasts. It is the folding together of ecological and historical processes, the crossings of bat migrations with “imperial debris” (Stoler 2008) that create the conditions for risky commensality. The hotspot opens up the “transmission event” to the elaborate temporal and material relationalities that cultivate networks of pathogenic exchange.

## Outbreak: Hospitals as Contexts for Viral Control and Amplification

During an outbreak of VHF, the problem for disease managers is to reorder human–human and human–object proximities to prevent the spread of the virus, while also providing care and treatment to sufferers.^15^ Transmission between humans, either directly or via fomites, accounts for the majority of cases of VHF. Clinical settings are key sites for both VHF management and nosocomial outbreak (e.g., Fisher-Hoch 2005:129–130).

**Figure 3:**
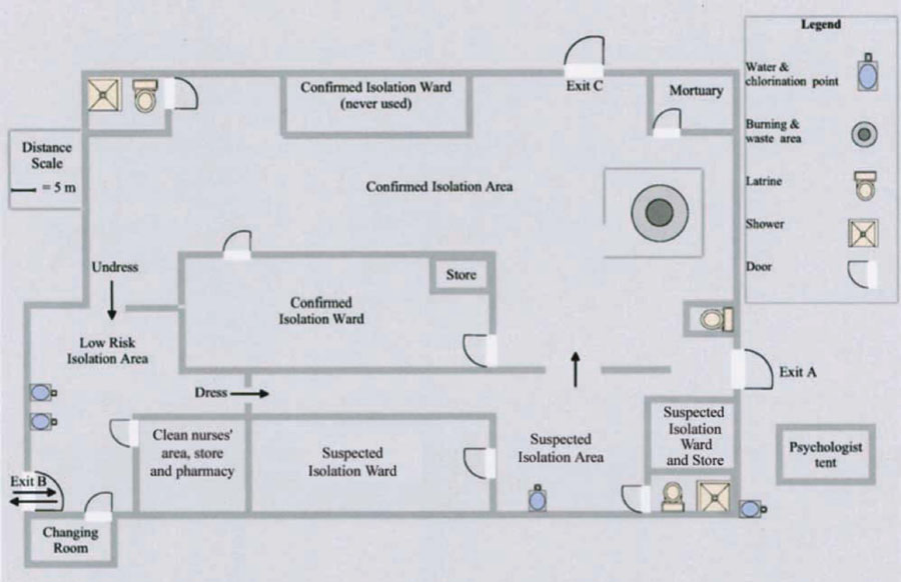
A diagram of the layout of the Marburg ward and isolation area near the end of the Marburg hemorrhagic fever epidemic, Uige, Angola, 2005 (Jeffs et al. 2007:196 Suppl. 2), indicating the highly spatialized nature of protection during an outbreak. Arrows indicate the movement of health personnel, patients, and corpses.

For some time, hospital ethnographers have criticized understandings of hospitals as sites formed primarily by the culture of biomedicine. Building on post-structuralist spatial logics (e.g., Ingold 2000; Massey 2005), they have developed rich descriptions of the diverse socio–spatial practices that shape medical institutions (Brown 2012; Street and Coleman 2012). Considered alongside recent anthropological analyses of *materia medica* (e.g., Whyte et al. 2002), these resources help underline the relevance of recent theoretical approaches in anthropology for understanding the dynamic encounters between people, institutional spaces, and nonhumans that can transform hotspots into sites for viral amplification (see Figure 3).

Moving beyond a notion of the hospital as a space within which ever-more protective barriers are erected to distance clinical care from everyday life, a logic exemplified by the image above, a spatialized ethnographic engagement draws attention to the divergent practices of care and protection that shape institutional spaces. As Alain Epelboin (e.g., Epelboin et al. 2005) has shown, situating the technical immediacies of VHF care, protection, and intervention in broader relational and social context can contribute to improving the management of VHF outbreaks. Placing such insights within fine-grained ethnographic accounts of the institutional histories and symbolic meanings of clinical spaces would further nuance our understanding of transmission dynamics.

Understandably, high numbers of hospital fatalities, the isolation of infected patients, and the use of full-body protective garb can produce considerable unease. In a number of outbreak situations, patients fled hospitals and refused to refer loved ones for treatment (Guimard et al. 1999). Partly in response to this sort of behavior, a central theme of the social–scientific literature on secondary transmission has been to reflect on the lessons learned from past outbreaks and to restore faith in hospitals as sites of emergency care. Some studies have focused on the challenges of disease management, communicating health warnings, monitoring cemeteries, and constructing isolation wards (e.g., Jeffs et al. 2007; Roddy et al. 2007), often drawing attention to the rumors that circulate around interventions and impede public health and hospital-based attempts to manage epidemics (Borchert et al. 2007:Appendix; Boumandouki et al. 2005; Hewlett et al. 2005; Roddy et al. 2007; Vives 2003).^16^ Others have explored the experiences of people such as health workers, survivors, and affected communities. Surveys, focus groups, and structured interviews have been used to ascertain the perspective of these people as well as experiences of stigma faced by those who survive (De Roo et al. 1998; Guimard et al. 1999; Hewlett and Amola 2003:1246).^17^

Much of this social science literature reinforces an analytical separation between the community and the hospital. Like research on bushmeat consumption, community-focused research has prioritized local beliefs and practices, detailing disease classification and burial practices (see also Boumandouki et al. 2005; Lamunu et al. 2004:31). Hospital-based research, in contrast, has focused on infection control and isolation, including fears of contagion and the protocols of hospital burial (Guimard et al. 1999; Jeffs et al. 2007; Lamunu et al. 2004; Okware et al. 2002). Only a very small number of accounts describe the clinical management of outbreaks from the perspective of the international scientists organizing these interventions (Guimard et al. 1999; Jeffs et al. 2007; Kerstiën and Matthys 1999) or the indigenous health workers providing patient care (Borchert et al. 2007; Guimard et al. 1999).

Clearly, the management of Ebola outbreaks within emergency wards leaves less room for anthropological analysis than public health campaigns focused on prevention. However, bringing ethnographic insights from the community and the hospital together illuminates practices that transcend the boundaries of the hospital, such as the care provided by family members within hospitals, or the practices of local health workers who belong to both domains. Even the highly guarded boundaries of the isolation ward are porous to social practices, even if not (ideally) to pathogens.

Existing work in medical anthropology, especially around the material artifacts of institutionalized medicine, provides a powerful example of the potential insights gained from an ethnographic sensibility to the co-production of medical and social contexts. For example, medical anthropologists have documented the ways in which the use of protective materials (such as gloves) in hospital settings can be influenced by a range of factors including prior relationships, fear of infection, and the desire to appear to be a modern medical practitioner. Work that one of the authors carried out in Kenya (Brown 2012:22) documented that, when in short supply, protective gear was sometimes used for long periods of time, and to care for many patients, thus protecting health staff but increasing the risk of infection. Meanwhile, health workers managing a Marburg epidemic in Uige, Angola, were less inclined to wear protective clothing when nursing family members and fellow health workers than when nursing other kinds of patients, despite awareness of the risks involved (Borchert et al. 2007). Elsewhere, Raabe et al. (2009) have explored the acceptability of different kinds of protective material used in VHF outbreaks—including body bags—in relation to indigenous concerns about visibility and care.

Anthropological insights are useful for understanding such distinctions and situating them within broader concerns around the management of the sick body. Jeffs et al. (2007:S158), for instance, describe the effect of the introduction of intravenous fluids during a Marburg outbreak in Uige, Angola:

Not only did they [the intravenous fluids] appear to improve survival, but they also appeared to greatly improve the patients’ and their families’ perceptions of the Marburg ward, which enhanced MSF's standing in the community. Patients expect injections and IV treatment in this area of Angola, and the number of individuals presenting to the Marburg ward for assessment increased after these treatment measures were introduced.

Such insights resonate with anthropological observations about perceptions of effectiveness associated with different kinds of medicines and medical interventions (e.g., Langwick 2007; Whyte 1982:2060; Whyte et al. 2002). In many African contexts, intravenous treatment is reserved for the most severe types of sickness; one of Brown's informants recently recounted the severity of a recent illness by repeatedly emphasizing the number of bottles of rehydration fluid (*maji*) she was given intravenously. Thus, it is not surprising, from an anthropological perspective, that people often expect IV treatment for an infection as serious as VHF or that they might associate its provision with the delivery of proper care.^18^

Our program for future research extends these analytical frameworks. Conceptualizing the hotspot in the hospital gives empirical priority to the ways in which clinical spaces and objects dynamically emerge through medical encounters. Like the monkey park, hospitals are a composite of diverse and divergent interactions; the materiality of medical therapies, instruments, or diseases is brought into being through diverse socio–spatial practices (Mol 2002:5). Take gloves for example: A standard tool of barrier nursing, gloves are used to prevent contamination of caregivers and patients; their effectiveness hinges on their rapid disposal. But in situations where the availability of medical instruments is erratic, gloves continue to perform modern care and appropriate practice, even when used repeatedly. Further, when nursing a family member or even a friend, the restraints gloves place on intimacy can be more important than the barriers they provide against contagion; during these interactions, they become something else entirely—a form of social distance, a mechanism of detachment.

Because gloves—like many medical objects—are enacted within relationally dense and profoundly affective practices, they project a multiplicity of different imaginaries, meanings, and uses, often simultaneously. Such meetings of radically different realities are not, by definition, problematic. However, this ontological multiplicity can create opportunities for viral transmission. As in the case of the twin village, it is in its capacity to mark out the emergence and convergence of difference within a shared landscape—or in this case, within the same object—that the hotspot gains its analytical traction.

## Material Proximities: Giving the Hotspot Ethnographic Traction

Today, much interpretive work in medical anthropology, particularly that conducted in the “global south,” is directed toward situating affliction within geopolitical and economic processes. Structural violence has provided a theoretical frame to explore those links as anthropologists attempt to show how the “social machinery of oppression” plays out in everyday life (Farmer 2004:312). Demonstrating that VHF outbreaks are a consequence of intersections between infected animals and colonial pasts is a central ethnographic task. Yet, as a means to render visible the diverse social, political, and material conjunctions on which transmission depends, structural violence has considerable limitations. Although it clarifies the interplay between macro forces and micro events, the concept elides the materiality of pathogenic encounters by limiting transmission processes to questions of economic vulnerability. Nor does it capture the varied agencies and interactions that connect deep structure to local experiences.

This is where the hotspot provides analytical mileage. The concept captures the complex relationality of VHF: the heterogeneous interactions between individuals, populations, and environments that lead to an outbreak. It elaborates these contextures across multiple scales, but without flattening them into a static network of connection. The hotspot denotes a thickening of fields; a layering of the relational possibilities and intensities that occasion transmission. For what is at issue is not simply that there are many different factors—ecological, economic, social, and political—that trigger and shape the spread of disease. Rather, outbreaks are the product of latent relations, the contingent convergence of pathogenic potentials. The hotspot does not stabilize an outbreak narrative but rather draws attention to the sudden, ephemeral, and material concurrences between humans, animals, non-humans, institutions, and pasts that occasion contagion. It is not a means of prospectively or retrospectively specifying the factors that might lead (or have led) to transmission, but a way of alerting us to the radical and contingent relationality through which outbreaks emerge.

An anthropology of the hotspot would explore viral movement by attending to the multiple material, historical, and social forms of connection brought about through closeness, contiguity, and propinquity. Here we draw inspiration from Joost Fontein (2011), who develops the concept of “material proximities” to probe the relationships between the materiality of particular places and distinct configurations of belonging and autochthony in post-colonial Zimbabwe. Rather than focusing on difference through an analytic of separation—between local politics and imperial policies, say, or village life and commercial development—Fontein (2011) takes up burial grounds, and the white and African ghosts that haunt them, to explore how distinct histories and representations co-exist within a landscape:

Just as graves and ruins around Mutirikwi are not inert material expressions of politically deployed languages of belonging and authority, but rather are active and affective in complex ways, so we should envisage historical, material, and conceptual proximities as involving active, changing engagements between peoples, things, epistemologies, and even ontologies. (Fontein 2011:722)

A heuristic of proximity is clearly fundamental to understanding viral transmission. Fontein's interest in proximity, however, is more intimate than contiguity—a state of being in physical contact. In his analysis, legacies of eviction and dispossession—of both white and black farmers—are entangled with diverse claims to ancestral authority; different pasts are co-present in the ruins of regimes, often in the same sites and objects. Difference is not manifest as a radical separation but emerges through a variety of material entanglements and coexistences within a shared landscape.

Attention to how radical forms of difference play out in the hotspot points to some of the connections neglected by conventional analysis—consider, for instance, that the outbreak ward is formed both through the construction of hyper-protective barriers and obligations to provide loving care; that abandoned plantations are remnants of declining colonial economies and sites of new hunting practices and markets for bushmeat. It is often at occasions when such differences become materially proximate that transmission occurs.

Critical too, are the spatial, temporal, and affective dynamics of these proximities: just as graves shape the contemporary politics of land in Zimbabwe, so the hotspot draws attention to the co-presence of divergent histories within sites of VHF transmission and management. The intersections between colonial remainders, independence policies, public health practice, and the emotive registers that inflect practices of care and support have tremendous significance for the pathogenicity of a milieu.

The distinct human, animal, political, and institutional practices that shape hotspots mark out both the differences of these arenas and their entangled material and historical proximities. The purpose of researching the hotspot ethnographically is not to arrive at a more precise explanation of an outbreak—a more complete picture of how, where, and when a virus moved across bodies and space. Rather, in eschewing the assumption of a stable and singular context of disease, an ethnography of the hotspot would elucidate the interactivity of pathogenic things and places through processes of transmission.

## Conclusion

Hotspots are places defined by excess and lack, the absence of resources and an abundance of pathogens. Here—as in many other public health contexts—anthropological research is carried out with the purpose of interrupting, uprooting, and undoing the density of these connections by drawing attention to the institutional politics, histories of violence, and intimate entanglements that bring them together.

In this article, we have sought to mark out a terrain for future ethnographic engagements with VHF by drawing on insights from multispecies ethnography, studies in material culture, and post-structuralist understandings of space. Through greater attention to the social and material interpenetration of “risky” spaces—hospitals, homes, the bush, the market—during and outside of outbreak situations—we can develop richer accounts about the spread of VHF across sites and scales of encounter. The theoretical concept of the hotspot provides a heuristic to explore the divergent and complex forms of material proximity that encourage viral movement, and is indicative of ways in which these different anthropological conversations might be made relevant to public health.

Finally, by bringing together sites and practices traditionally held apart within and beyond anthropology, the hotspot opens up possibilities for new forms of collaboration between anthropologists, ecologists, and disease managers. Conceptualizing expertise as falling into discrete domains—bat bodies and ape behavior, the genetic make-up of a virus and the rate of deforestation, hunting practices and hospital hygiene—has considerable limitations. We hope that extending the ethnographic scope of engagements with VHF will also enable a rethinking of the terms of interdisciplinary exchange. A robust multi-dimensional approach to public health interventions rests on drawing together distinct ways of knowing rather than integrating different objects of knowledge. This collaborative zone is, perhaps itself a hotspot, where ideas can move between hosts as they approximate (or make proximate) their differing knowledge practices.

## Notes

1 Viral hemorrhagic fevers are caused by one of four families of RNA viruses—Arenaviridae, Filoviridae, Bunyaviridae, and Flaviviridae. These viruses are found around the globe in a number of different forms. This article concentrates on African strains of Ebola, Marburg, and Lassa fever. Although these share some key epidemiological and ecological characteristics (e.g., the role of small mammals in hosting the pathogen and of hospital settings in viral spread), our focus follows from the research objectives outlined by a multidisciplinary consortium on which we had the opportunity to collaborate, thus it reflects the analytic scope of this project and the interests of our collaborators. We hope that this article will lead to development of a methodological approach that will help open up these categorizations for reflection and reconfiguration.

2 In 1999, the Centers for Disease Control and Prevention (CDC) classified VHF as high-priority (Category A) bioweapon agents, determined by their ease of dissemination, potential public health impact, and requirement of special requirements containment (http://www.bt.cdc.gov/Agent/Agentlist.asp).

3 With the exception of Lassa, which is endemic in a number of West African countries, VHF infections are rare. When outbreaks do occur, the virulence of these diseases inhibits widespread transmission, as the majority of sufferers die before they can infect others (M. Borchert, personal communication). Despite this epidemiological reality, social theorists have argued that these diseases are emblematic of the new and unruly connectivities (ranging from terrorism to economic crises) that shape national security concerns and prompt imperialistic approaches to global health (Clarke 2013; Hinchliffe and Bingham 2008).

4 In particular, our reflections stem from the ways in which the distribution of research was imagined by the collaboration: Theme one focused on the viral reservoirs of VHF; theme two, the practices and situations that might bring people into contact with those reservoirs; and theme three examined the human to human transmission of the virus. We were granted considerable freedom to describe the nature of an anthropological contribution, though there were some key objects (e.g., hunting practices, burial rituals) that were expected to seize our attention. This article takes both that empirical latitude and that collaborative spirit seriously by aiming to extend ethnographic engagement across the three themes.

5 Thinness is a measure of interpretive depth and not empirical accuracy; a “thick” description elaborates the nuances of cultural meaning on a small scale; it “grows out of the delicacy of its distinctions not the sweep of its abstractions” (Geertz 1973:25).

6 This approach is characteristic of the One Health Agenda that seeks to “enhance cooperation and collaboration between physicians, veterinarians, other scientific health and environmental professionals” (http://www.onehealthinitiative.com/about.php).

7 This approach shares some concerns with Rappaport's work (e.g., 1996) in anthropological ecology. However, where Rappaport argues for an expanded understanding of environment through his concept of human systems, our concern is with the particular way in which the social is conceptualized.

8 See CDC: http://www.cdc.gov/ncidod/dvrd/spb/mnpages/dispages/lassaf.htm.

9 Airborne transmission has been observed in the lab, though not yet documented in a real-world setting (CDC 2009).

10 Lassa fever can be treated with an intravenous administration of Ribavarin, but its success hinges on an early diagnosis, which, owing to varied symptoms and limited availability of reference laboratories in endemic countries, can be very difficult.

11 For instance, armed conflict not only disrupts local networks of trade, but also allows greater access to weapons that can be used to hunt (de Merode et al. 2004); commercial logging has not only fragmented habitats, but also created the infrastructure to enable the transportation of meat over wide distances (Karesh and Noble 2009).

12 In a slightly different vein, anthropologists have also explored the connections between hunting, display of animals, and conservation as deeply embedded in the colonial enterprise (Haraway 1989; MacKenzie 1988).

13 For instance, the genetic sequencing of HIV strains suggest that the virus had been transmitted from primates to humans a number of times in the last century before the virus emerged globally (Karesh and Noble 2009).

14 The importance of asymptotic infections resonates with Gonzales et al.'s 2010 recuperation of Mirko Grmek's term “pathoecenosis” to capture the interactivity of infectious agents within a given territory across human and animal species (Grmek 1998).

15 Lassa fever, endemic in parts of Sierra Leone, does not fully correspond to what (Leach and Dry 2010) have called the “outbreak narrative” characteristic of other VHFs like Ebola and Marburg. However, Lassa can produce epidemic exacerbations (often through hospital amplification), and involves the mobilization of new forms of hospital-based care (e.g., Donaldson 2009). Further, like Ebola and Marburg, the virus is present in animal hosts and “jumps species” through particular kinds of human–animal interaction.

16 However, this literature has largely failed to engage with broader social science literature on rumor and medicine in Africa (e.g., Geissler 2005; White 2000).

17 This research is often circumscribed to the locales where public health work takes place. For instance, Merlin, the U.K.'s leading international health charity and responsible for managing Lassa fever during the Sierra Leonean civil war (Kirkland 2003; Merlin 2002a, 2002b, 2002c), produced detailed accounts of transmission drawn from their base of operations, the town of Kenema.

18 Fears that use of IV medications would increase the risk of transmission to health professionals discouraged the use of IV fluids in many VHF epidemics. However, the failure to provide treatment recognized as constituting good care became viewed in a number of settings as a policy of letting people die, with Ebola wards labeled as “death houses” in some contexts (M. Borchert, personal communication).
